# Temperature and Humidity Sensor Powered by an Individual Microbial Fuel Cell in a Power Management System

**DOI:** 10.3390/s150923126

**Published:** 2015-09-11

**Authors:** Qi Zheng, Lei Xiong, Bing Mo, Weihong Lu, Suki Kim, Zhenyu Wang

**Affiliations:** 1 College of Food Science and Engineering, Harbin Institute of Technology, Harbin 150001, China; E-Mails: mbzq@163.com (Q.Z.); lwh@hit.edu.cn (W.L.); 2 Department of Electrical Engineering, Huaqiao University, Xiamen 361021, China; E-Mails: ydrxbqxl@163.com (L.X.); mobing@hqu.edu.cn (B.M.); 3 Department of Electrical Engineering, Korea University, Seoul 361021, Korea; E-Mail: skkim@korea.ac.kr

**Keywords:** microbial fuel cells (MFCs), power management system (PMS), sensors, charge pump, microcontroller unit (MCU), boost converter, DC-DC

## Abstract

Microbial fuel cells (MFCs) are of increasing interest as bioelectrochemical systems for decomposing organic materials and converting chemical energy into electricity. The main challenge for this technology is that the low power and voltage of the devices restricts the use of MFCs in practical applications. In this paper, a power management system (PMS) is developed to store the energy and export an increased voltage. The designed PMS successfully increases the low voltage generated by an individual MFC to a high potential of 5 V, capable of driving a wireless temperature and humidity sensor based on nRF24L01 data transmission modules. With the PMS, MFCs can intermittently power the sensor for data transmission to a remote receiver. It is concluded that even an individual MFC can supply the energy required to power the sensor and telemetry system with the designed PMS. The presented PMS can be widely used for unmanned environmental monitoring such as wild rivers, lakes, and adjacent water areas, and offers promise for further advances in MFC technology.

## 1. Introduction

With increasing attention being paid to global warming and the depletion of fossil fuels, substantial investment and effort has been aimed toward developing renewable energy technologies. Microbial fuel cells (MFCs) represent a novel alternative power source, utilizing organic energy for the production of electricity. Energy generation based on MFCs offers bright prospects as an environmentally friendly energy source because of their mild reaction conditions and the wide availability of suitable fuel [1]. Although reactors based on MFCs can develop an open circuit voltage of approximately 1.1 V [2], a more typical voltage is 0.5 V, which is insufficient to drive most practical sensors and electrical components. Nevertheless, some researchers have designed energy harvesting circuits that can be powered by the lower output of MFCs [3,4]. Gong *et al*. have use super capacitors and a boost converter to power the oxygen and temperature sensor; however, this system requires an additional battery [5]. Hatzell *et al*. have used multiple MFCs connected in series to increase the output voltage [6]; however, this method is rather complex for practical applications because multiple cascaded MFCs are difficult to construct and may result in voltage reversal [7,8,9]. Therefore, the study presented in this paper used an individual MFC as the power source to drive the load with the help of the designed circuit.

In this paper, a power management system (PMS) is designed to harvest energy based on a charge pump with the help of a supercapacitor as an energy storage element. When the PMS starts operating, the supercapacitor accumulates energy in small increments during the charge period, and discharges it to a DC-DC boost circuit during the discharge period. The DC-DC boost circuit generates a 5 V voltage to simultaneously power a temperature and humidity sensor. With the assistance of the cyclical operation of this PMS, an external load can be powered by an individual MFC. 

For this study, a wireless sensor is added at the load end of an nRF24L01 data transmission module. A PMS is needed to export the 5 V output voltage from an individual MFC to enable the sensors to capture the temperature and humidity data. Microcontroller units (MCUs) obtain the data and transmit them using a wireless module. Computers can acquire the information from the sensors at the receiving end using serial ports. The computers use a serial port software based on a virtual instrument, enabling the display and storage of data and the communication between the MCUs and computers.

This study demonstrates that the designed circuit has realized the function of gathering the steadily accumulating energy produced from an individual MFC and using this energy to drive the sensor and telemetry system. To confirm functionality, a remote sensing of temperature and humidity experiment was conducted using a DC-DC boost circuit with the aim of increasing the voltage and power availability. The circuit shown in this article will allow wider adoption of MFCs and should find wide application in the unmanned environmental monitoring field.

## 2. Materials and Methods

Section 2.1, Section 2.2, Section 2.3 and Section 2.4 describe the operational mechanism of MFCs, the proposed PMS, the sensor and telemetry system used to test the functionality of the PMS, and the sensor’s program design, respectively.

### 2.1. Operational Mechanism of MFCs

#### 2.1.1. Mechanism of Electricity Production by MFCs 

Dual-chamber MFCs are commonly used in the laboratory; they consist of an anode chamber and a cathode chamber separated by a proton exchange membrane (PEM). The anode chamber provides an anaerobic environment that is beneficial to the growth of microorganisms. The metabolism of the microorganisms oxidizes the organic substance and transfers electrons to the anode; these electrons then traverse the external load to the cathode resulting in the production of a current. At the same time, protons from which water is produced from the reaction of oxygen with the protons pass through the PEM to the cathode. As has been found in many surveys, the electrode materials, the space between the electrodes, and the density of the substrate all affect the efficiency of electricity production by MFCs [10,11,12,13,14,15,16].

#### 2.1.2. MFC Setup and Operation.

For this study, the dual-chamber cubic MFC was used, with each chamber having an empty volume of 27 mL. Both the anode and cathode employed a carbon felt material, each with a projected surface area of 9 cm^2^ (3 cm × 3 cm), as electrode materials upon which microorganisms can easily adhere, thus producing more electrons with more rapid migrations to the electrodes. The reason for selecting the large area of the electrodes is that the large area allows a more convenient transfer of electrons, and the internal resistance is reduced accordingly. 

The MFC was inoculated with an anaerobic mixed culture collected from a wastewater treatment plant. The anode chamber was filled with artificial wastewater containing CH_3_COONa (with density of 0.823 g/L), Na_2_HPO_4_·12H_2_O (with density of 11.46048 g/L), and NaH_2_PO4·2H_2_O (with density of 2.80818 g/L). A phosphate-buffered K_3_[Fe(CN)_6_] solution containing K_2_HPO_4_·3H_2_O (with density of 7.3 g/L), KH_2_PO_4_ (with density of 2.44 g/ L), and K_3_[Fe(CN)_6_] (with density of 16.46 g/L) was used as a catholyte to minimize the cathode effects on system performance. 

To start the MFC, 5 mL of inoculum and 20 mL of artificial wastewater was injected into the anode chamber using a syringe. Originally, the reactor is run in a batch-fed mode and is manipulated until repeatable voltages are achieved at the external resistor of 1 kΩ, for the MFC at room temperature. Subsequently, the reactor is run in a continuous-flow operation by exchanging 20 mL of fresh artificial wastewater with 20 mL of depleted anolyte in each MFC.

### 2.2. Design of the PMS

**Figure 1:**
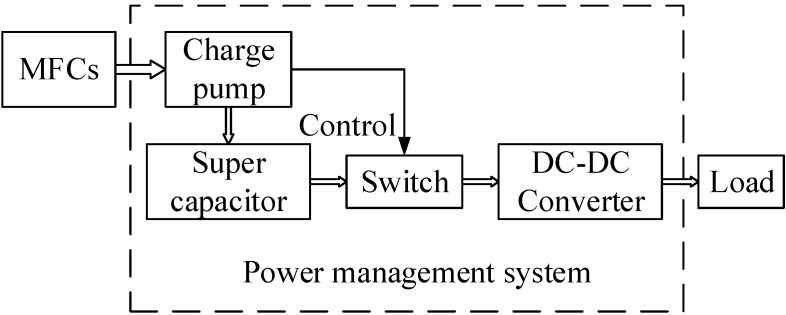
Schematic of the PMS.

The proposed PMS shown in Figure 1 is composed of a charge pump, a supercapacitor, a switch, and a DC-DC converter. The charge pump is used to store the energy produced by the MFCs in the supercapacitor until sufficient electricity has been accumulated to provide an initial voltage for the boost circuit. The supercapacitor was employed as the storage component because it can store a higher energy density than a conventional capacitor over the short charging time. When the voltage increases to a set threshold value, a control signal is generated from the charge pump that closes the switch between the supercapacitor and the DC-DC converter, and the supercapacitor begins to discharge. The switch remains off until the voltage of the supercapacitor drops below another set value. In this way, the charge pump acts as a switch to connect and disconnect the supercapacitor with the load. The equivalent input resistance of the charge pump should be proper to make the MFCs achieve reasonable electricity production performance. There are some reports on harvesting energy at the MFC’s maximum power point [17,18,19]. However, all the reported maximum power point tracking circuits require batteries as an additional power supply. To drive sensors with an individual MFC without any additional power, this study pursues the appropriate power point by using a fixed value supercapacitor rather than the maximum power point tracking circuits. This study uses the 3 F value as the best value of the supercapacitor for achieving the desired result, based on our previous testing results. The proposed system aims to accumulate the energy generated from the MFCs, and then use the accumulated energy to power the external circuit through the DC-DC converter.

**Figure 2:**
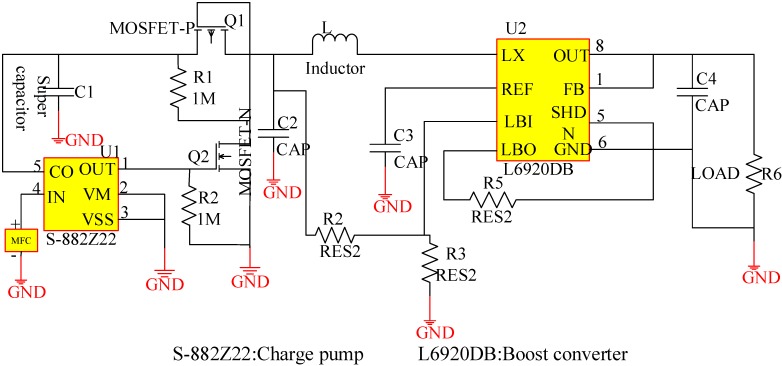
Energy harvesting circuit based on the charge pump and supercapacitor.

The energy harvesting circuit has been implemented using an individual MFC as the power source. As shown in Figure 2, the positive input of the MFC can be attached to the charge pump (S-882Z22). A 3 F super capacitor (C1 in Figure 2) is capable of storing energy to accumulate electricity and control the switch. The N-channel MOSFET (Q2 in Figure 2) and P-channel MOSFET (Q1 in Figure 2) are used as the two switches. When the voltage of the supercapacitor’s positive electrode reaches the value of 2.2 V, the charge pump exports 2.2 V at the OUT node, and then closes the Q2 switch when the voltage of Q2 drain node has been drained to almost 0 V. Because the Q1 gate node is connected to the Q2 drain node, switch Q1 is also closed at this time. Therefore, the C1 supercapacitor discharges to the DC-DC boost circuit (L6920DB), which can increase the input voltage of 2.2 V to an output voltage of 5 V. When the voltage of C1’s positive electrode drops below 1.6 V, the charge pump outputs a low level, which opens switch Q2, resulting in the disconnect of the Q2 drain node from the ground node and a sudden increase in the voltage to almost 1.6 V. Subsequently, the switch Q1 opens and the MFC again begins to charge the C1 supercapacitor. 

**Figure 3:**
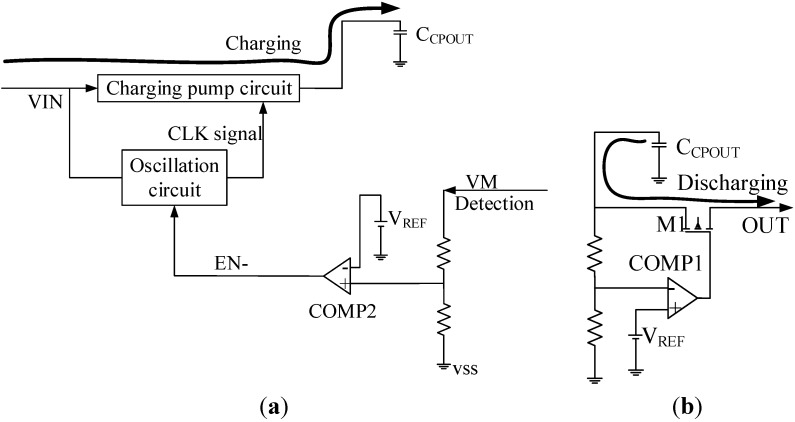
Schematic of S-882Z22. (**a**) Principle of charging mode; (**b**) Principle of discharging mode.

A low input voltage can satisfy the charge pump’s drive demand when using S-882Z22, which can operate normally with an input of 0.3 V, 0.5 mA. The functions of the charge pump’s pins are described in Table 1 [20]. The schematic circuit diagram of S-882Z22 is shown in Figure 3 [20].

**Table 1:** Functions of the charge pump’s pins [20].

Pin No	Pin Name	Pin Description
1	OUT	Output pin (step-up DC-DC converter connection pin)
2	VSS	GND pin
3	VM	Step-up DC-DC converter output voltage monitor pin
4	VIN	Power supply input pin
5	CPOUT	Startup capacitor connection pin

The schematic circuit diagram of S-882Z22 is shown in Figure 3. The power supply is connected to S-882Z22 through the pin Vin, and the external supercapacitor C_CPOUT_ is used as the energy storage element [20]. In this study, discharge start voltage and stop voltage are 2.2 V and 1.6 V respectively, the VM pin is connected to the VSS pin to ensure that the charge pump circuit operates all the time, preventing malfunction of the PMS. 

The L6920DB is a high efficiency monolithic step-up switching converter integrated circuit (IC) especially designed for battery-powered applications. The start-up voltage of the L6920DB is 0.8 V, and its input voltage can be as high as 5.5 V, with current as high as 750 mA. The output voltage can be adjusted, ranging from 1.8 to 5 V. The pin description for the L6920DB is shown in Table 2 [21].

**Table 2:** Pins description for L6920DB [21].

Pin No.	Pin Name	Pin Description
1	FB	Output voltage selector. Connect FB to GND for Vout = 5 V or to OUT for Vout = 3.3 V. Connect FB to an external resistor divider for adjustable output voltage
2	LBI	Battery low voltage detector input. The internal threshold is set to 1.23 V. A resistor divider is needed to adjust the desired low battery threshold.
3	LBO	Battery low voltage detector output. If the voltage at the LBI pin drops below the internal threshold typ. 1.23 V, LBO goes low. The LBO is an open drain output and so a pull-up resistor (about 200 KΩ) has to be added for correct output setting
4	REF	1.23 V reference voltage. Bypass this output to GND with a 100 nF capacitor for filtering high frequency noise. No capacitor is required for stability
5	SHDN	Shutdown pin. When pin 5 is below 0.2 V the device is in shutdown, when pin 5 is above 0.6 V the device is operating.
6	GND	Ground pin
7	LX	Step-up inductor connection
8	OUT	Power OUTPUT pin

The switches used in the circuit are N-channel MOSFET (Si3460) and P-channel MOSFET (Si3499), with thresholds between 0 and 0.6 V. The working threshold changes with the temperature, with a threshold of approximately 0.6 V at a room temperature of 25 °C. 

### 2.3. Sensor and Telemetry System

**Figure 4:**
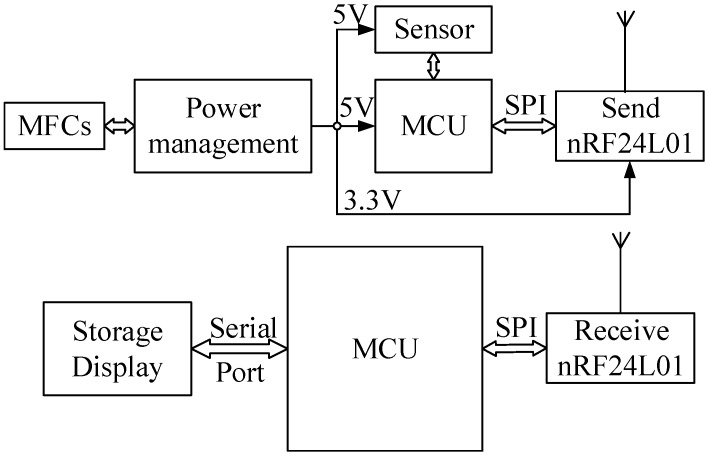
Schematic of the sensor and telemetry system driven by the MFCs.

One transmitter and one receiver, shown in Figure 4, constitute the sensor and telemetry system. The energy produced from the MFCs has been administered by the PMS so as to increase a low potential to a high potential with a value of 3.3 or 5 V, which can drive the transmission system. The temperature and humidity sensors and the microcontroller unit (MCU) require a working voltage of 5 V, whereas the wireless transmitting module nRF24L01 requires 3.3 V. The function of the MCU is to obtain and transmit the data from the sensor. It is necessary for the receiver to use serial ports, through which the data can appear on a computer monitor while also storing the data in the computer. 

#### 2.3.1. Transmitter of the Sensor and Telemetry System

The hardware of the transmitter consisted of sensors, a microcontroller unit (MCU), a wireless module, and the connections at communication interfaces among the different kinds of circuit modules.

**Figure 5:**
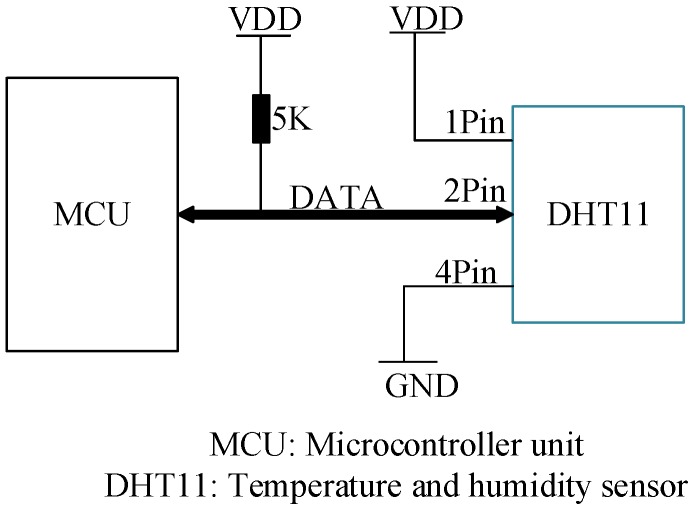
Application circuit of the temperature and humidity sensor.

In this study, an 8-bit low power MCU (STC89C52) was used to obtain and transmit the data from the temperature and humidity sensor. The DHT11 temperature and humidity sensor used in this study can be connected to the MCU with only one data line, which conserves the interface resources. The pins description of DHT11 is shown in Table 3 [22]. The circuit scheme is shown in Figure 5.

**Table 3:** The pins description of DHT11 [22].

Pin No.	Pin Name	Pin Description
1	VDD	3 V to 5 V, powered directly
2	DATA	Serial data lines, signal bus
3	NC	Untapped pin, suspended
4	GND	Power negative ground

This work used a wireless transmission module (nRF24L01) as the transceiver, which works in the Industrial Scientific Medical (ISM) band from 2.4 to 2.5 GHz. The current consumption of the transmission module is relatively low at 11.3 mA at 0 dBm output power. If the system remains in standby and power down mode, the current consumption of the wireless transmission module is even lower at 900 nA in power down mode, 26 µA in standby-I mode, and 320 µA in standby-II mode [23]. Therefore, this wireless transmission module is appropriate for low power wireless applications. The pins description of nRF24L01 is shown in Table 4 [23].

**Table 4:** Pins description of nRF24L01 [23].

Pin No.	Pin Name	Pin Description
1	CE	Chip Enable Activates RX or TX mode
2	CSN	Serial Peripheral Interface (SPI) chip select
3	SCK	SPI Clock
4	MOSI	SPI Slave Data Input
5	MISO	SPI Slave Data Output
6	IRQ	Interrupt Pin
7	VDD	Power Supply (+3 V DC)
8	VSS	Ground(0 V)

Sensor DHT11 can capture the temperature and humidity data from the ambient environment, and the MCU detects the data for transmission. A serial peripheral interface (SPI) is used to transmit data with the wireless module nRF24L01. Because there is no hardware SPI in the MCU STC89C52, the IO (input and output) port of the MCU was used to simulate the timing sequence of the SPI. In this way, the data communication between the MCU and wireless module can be achieved. 

#### 2.3.2. Receiver of the Sensor and Telemetry System

The hardware of the receiver consisted of a MCU, wireless receiver module, voltage converter circuit, and the connections among communication circuits between the MCU and computer. In this study, conversion between USB and the serial port is implemented by a conversion module CH340T. CH340T is a full speed USB interface device, the peripheral components of which require only capacitors and a crystal oscillator. CH340T is also a full duplex communication serial interface with a built-in transceiver buffer. The communication Baud rate ranges from 50 bps to 2 Mbps. The power supply voltage is either 5 V or 3.3 V. The pins description of CH340T is shown in Table 5 [24].

**Table 5:** The pins description of CH340T [24].

Pin No.	Pin Name	Pin Description
3	TXD	Transmitting data
4	RXD	Receiving data
6	UD+	USB data line D+
7	UD-	USB data line D-
9	XI	Linking with 12M crystal oscillator
10	XO	Linking with 12M crystal oscillator

The supply voltage of the wireless module is 3.3 V; however, the voltage generated from an individual MFC is increased by the DC-DC booster converter (AMS117) to 5 V. AMS117 is a low dropout voltage regulator that can implement the conversion between 5 and 3.3 V. 

### 2.4. Program Design of the Wireless Sensor 

**Figure 6:**
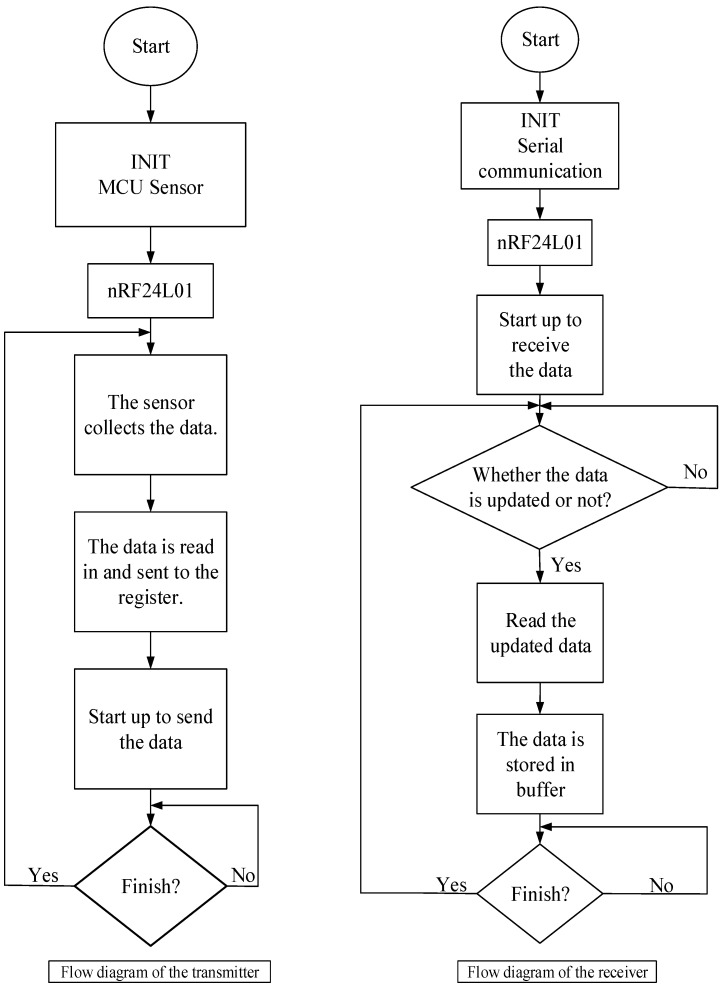
Flow diagram of the program.

The software design of the MFC energy harvesting system also consists of transmitting and receiving parts. In the transmitter part, the sensor DHT11 detects the temperature and humidity data and sends the data through the wireless module nRF24L01 to the MCU, which reads the data from the sensor. In the receiver part, the data can be stored in a buffer prepared for the communication between the MCU and computer. The flow diagram of the program is described in Figure 6.

#### 2.4.1. Program Design of the Sensor Collecting Data

The sensor DHT11 uses the signal bus with a bidirectional data-flow transmission format. The data line is used to synchronize and communicate between the sensors and the MCU. The data consists of integer and decimal parts with a total volume of 32 bit in the following format: 8 bit integer part of the temperature plus 8 bit decimal part of the temperature plus 8 bit integer part of the humidity plus 8 bit decimal part of the humidity. Firstly, the MCU sends a start signal. The sensor DHT11 changes from a lower power consumption mode into a high-speed operation mode. When the initial signal from the MCU ends, the sensor DHT11 transmits the response signal, which is a complete 32 bit signal. 

The users can read part of the data selectively. If the sensor cannot receive the initial signal transmitted from the MCU, the sensor cannot detect the temperature and humidity data. The sensor will revert to the low-speed working mode after data collection.

#### 2.4.2. The Program Design of the Wireless Module

The nRF24L01 can be configured in four main modes of operation: TX, RX, power down, and standby. The mode of operation depends on the registers CE, PWR_UP, and PRIM_RX, as shown in Table 6. By setting the PWR_UP bit in the CONFIG register to 1, the device enters standby-I mode. Standby-I mode is used to minimize average current consumption while maintaining short start up times. In this mode, only part of the crystal oscillator is active. Change to active modes only occurs if CE is set high; when CE is set low, the nRF24L01 returns to standby-I mode in both the TX and RX modes. In standby-II mode, additional clock buffers are active and more current is used compared to standby-I mode. The nRF24L01 enters standby-II mode if CE is maintained high on a PTX device with an empty TX FIFO (first in first out). If a new packet is uploaded to the TX FIFO, the PLL (Phase Locked Loop) immediately begins operation, and the packet is transmitted after the normal PLL settling delay of 130 μs. The RX mode is an active mode in which the nRF24L01+ radio is used as a receiver. To enter this mode, the nRF24L01 must have the PWR_UP bit, PRIM_RX bit, and tCE pin set high. The TX mode is an active mode for transmitting packets. To enter this mode, the nRF24L01 must have the PWR_UP bit set high, PRIM_RX bit set low, a payload in the TX FIFO, and a high pulse on the CE for more than 10 μs. Therefore, reasonable adoption of these different modes can reduce the power consumption of the whole circuit.

**Table 6:** Operation modes configuration [24].

Mode	CE	PWR_UP	PRIM_RX	Register FIFO (First In First Out)
TX mode	1	1	0	Data in TX FIFOs. Will empty all levels in TX FIFOs ^a^
TX mode	Minimum 10 μs high pulse	1	0	Data in TX FIFOs. Will empty one level in TX FIFOs ^b^
RX mode	1	1	1	/
Standby-I	0	1	/	No ongoing packet transmission
Standby-II	1	1	0	TX FIFO empty
Power down	/	0	/	/

^a^ in this operating mode, if the CE is held high, all TX FIFOs are emptied and all necessary ACK and possible retransmits are conducted. The transmission continues as long as the TX FIFO is refilled. If the TX FIFO is empty and the CE is still high, nRF24L01+ enters standby-II mode. In this mode the transmission of a packet is initiated as soon as the CSN is set high after an upload (UL) of a packet to TX FIFO; ^b^ In this operating mode, the CE pulses high for at least 10 μs. This is the normal operating mode, which allows one packet to be transmitted. After the packet is transmitted, the nRF24L01+ enters standby-I mode.

There are two methods for handling the data packet from nRF24L01: ShockBurst mode and enhanced ShockBurst mode. ShockBurst mode is typically used for simplex communications. Enhanced ShockBurst mode has the advantage of offering two-way transmission. The format of the data packet used in this study is shown as follows:
Preamble 1 byteAddress 3–5 byte Data 1–32 byteCRC 1–2 byte


The preamble is a bit sequence used to detect 0 and 1 levels in the receiver. An address ensures that the correct packet is detected by the receiver. The width of the cyclic redundancy check (CRC) can be set to 0, 1, or 2 bytes. The 1-byte CRC method has a higher data transmission efficiency. However, the 2-byte CRC method has better data integrity. In general, the data transmission speed via the wireless module is different from the MCU. The data handling speed of the MCU STC89C52 is less than the data transmission speed of the wireless module nRF24L01. Therefore, the transmitted signals from the lower MCU must be written in the wireless module FIFO to achieve the transportation of the data between the low-speed devices and the high-speed devices. 

**Figure 7:**
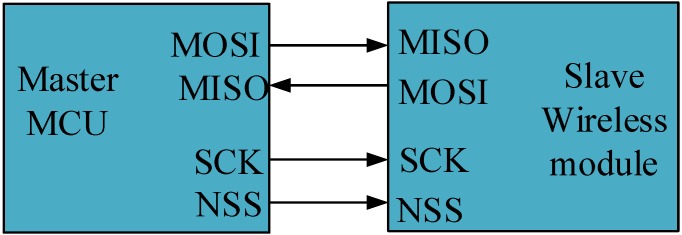
Communication method between the master device and the slave device.

The entire configuration of nRF24L01 is in a register configured as a SPI, which is a full-duplex communication interface that transmits the data synchronously. The maximum transfer speed of a standard SPI is 10 Mbps. The SPI enables serial communication among various pieces of peripheral equipment and the MCU. In general, the interface uses four lines: Serial Clock (SCK), Master Input/Slave Output (MISO), Master Output/Slave Input (MOSI), and Slave Selected (NSS). The internal hardware of the SPI used in this study consisted of two shifting registers, and the SPI can transmit the 8-bit data. There are two working modes for the SPI: master mode and slave mode. The communication method between the master device and the slave device is shown in Figure 7. This study used the MCU as the master device and the wireless nRF24L01 as the slave device. In the master mode, the SPI requires data transmission through the NSS and SCK lines, and the slave device receives data in the NSS and SCK lines. The register should be set to the initial value before the SPI begins data transmission. 

**Figure 8:**
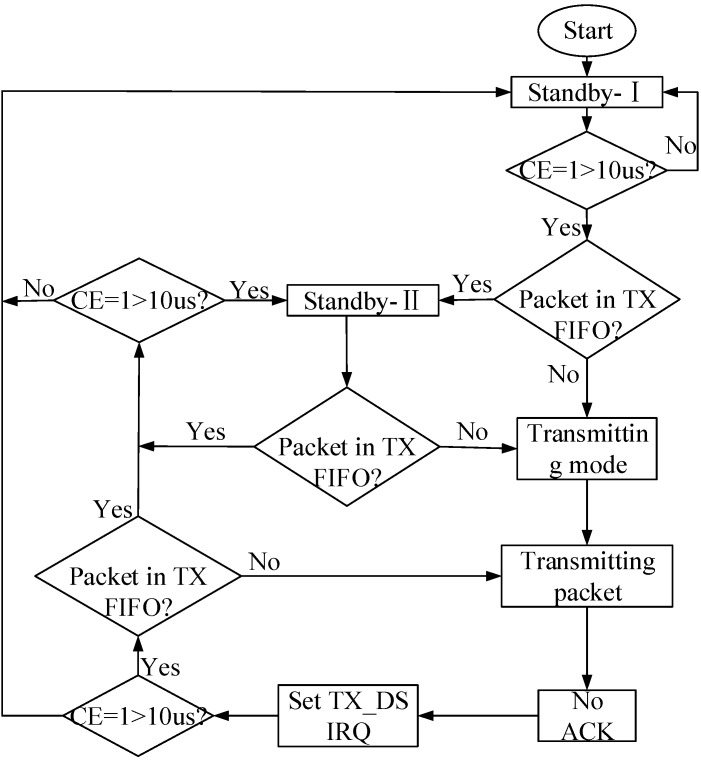
The nRF24L01 data transmission process.

In the nRF24L01 data transmission program, the wireless module remains in the transmitting mode when the CE is set to a high level and maintained for at least 10 s. The pins of the nRF24L01 data lines are set as input. The humidity data detected by the sensors is written through MOSI. Subsequently, the data is written in the TX_FIFO register of nRF24L01 and the CE setting changes from the high level to a low level. The wireless module goes into the ShockBurst mode of transmitting the data. The nRF24L01 data transmission process is shown in Figure 8. 

**Figure 9:**
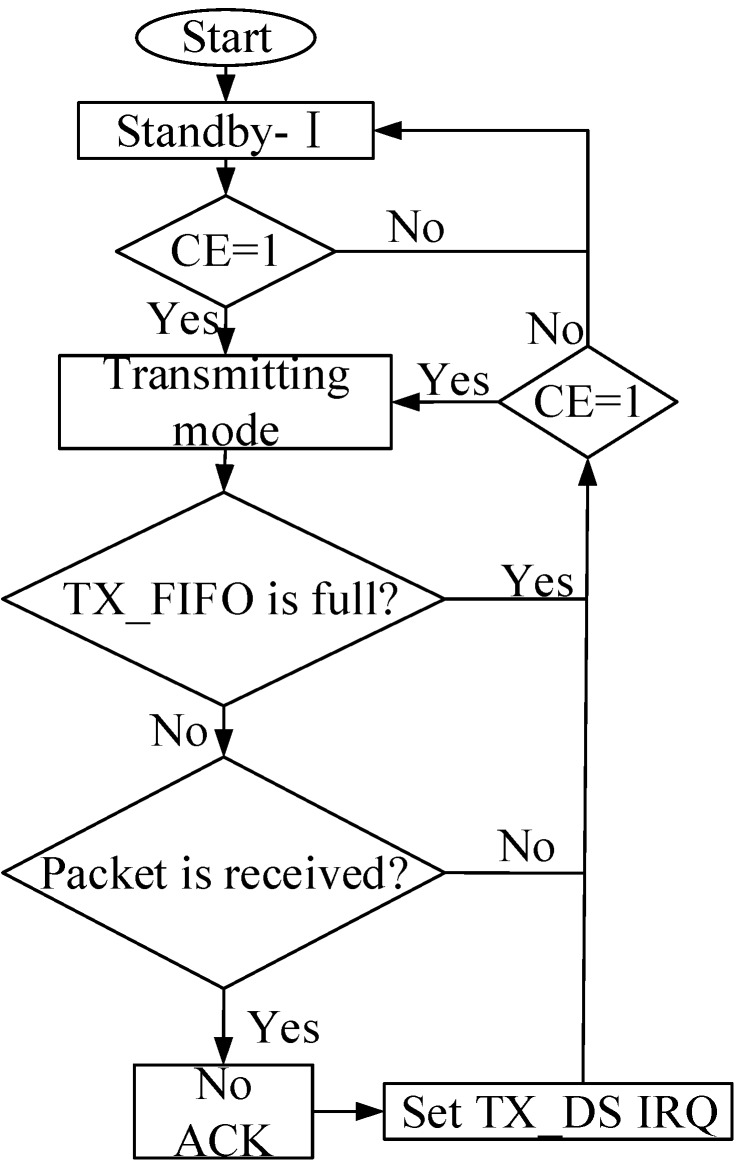
The nRF24L01 data reception process.

In the nRF24L01 receiver program, CE was set to 1, and PWR_UP was also set to 1. The configured CRC, the width of the address, the channel, and the transfer speed of the receiver should all be equal to those of the transmitter. The nRF24L01 data reception process is shown in Figure 9.

## 3. Results and Discussion

### 3.1. Characterization of the MFCs

**Figure 10:**
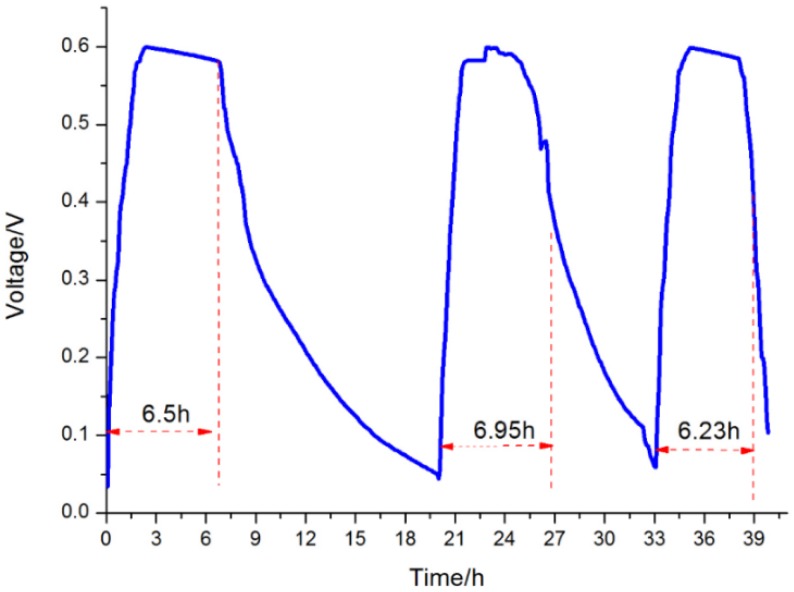
Three continuous flow cycles showing voltage variation at the external resistance of 1 kΩ.

In general, after starting the MFC, the open-circuit voltage (OCV) was measured by a digital multimeter (Keithley Instruments Inc., Cleveland, OH, USA). The OCV could reach 0.733 V. Subsequently, a 1 kΩ resistor was linked to the MFC. The beginning of a first period was defined as the point at which the output voltage approached 0.6 V; from this point, three stable periods were recorded by the same digital multimeter, as shown in Figure 10. From this procedure, it could be concluded that the voltage of the resistor was approximately 0.59 V, and each period lasted approximately 6.5 h. The steep decline in the voltage curve after the stable period of 6.5 h results from the exhaustion of the organic substance by the microorganisms. If wastewater in the anode chamber is refreshed, the output voltage could increase up to the stable value within a few minutes. In this study, 15 mL of wastewater is replenished at the end of each exhaustion period to the anode chamber, the empty volume of which is 27 mL; subsequently, approximately 0.59 V of output voltage from the MFC could be achieved in a few minutes. With 0.59 V as the output voltage and 6.5 h as the average time of every period, the current, power, and energy can be calculated as follows:

Current: I = U/R = 0.59 V/1 kΩ = 0.59 mA
Power: P = U^2^/R = (0.59 V)^2^/1000 Ω = 0.35 mW
Energy: W = P × t = 0.35 mW × 6.5 × 3600 s = 8.19 J

### 3.2. Energy Harvesting Test Results

**Figure 11:**
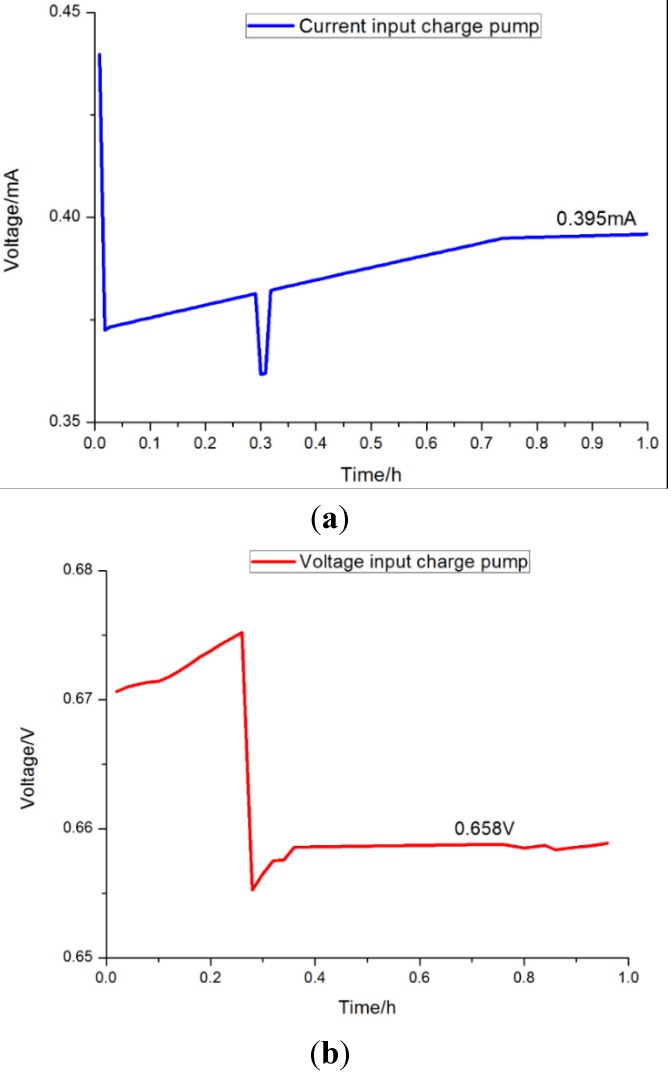
Voltage and current input through the charge pump. (**a**) Current input of charge pump; (**b**) Voltage input of charge pump.

Figure 11 shows the voltage and current input through the charge pump. As can be seen from the figure, the voltage remained at approximately 0.658 V after an initial drop. The current maintained a steady value of 0.395 mA. Using the charge pump, this energy was transferred from the individual MFC where it was generated to the supercapacitor for storage. 

**Figure 12:**
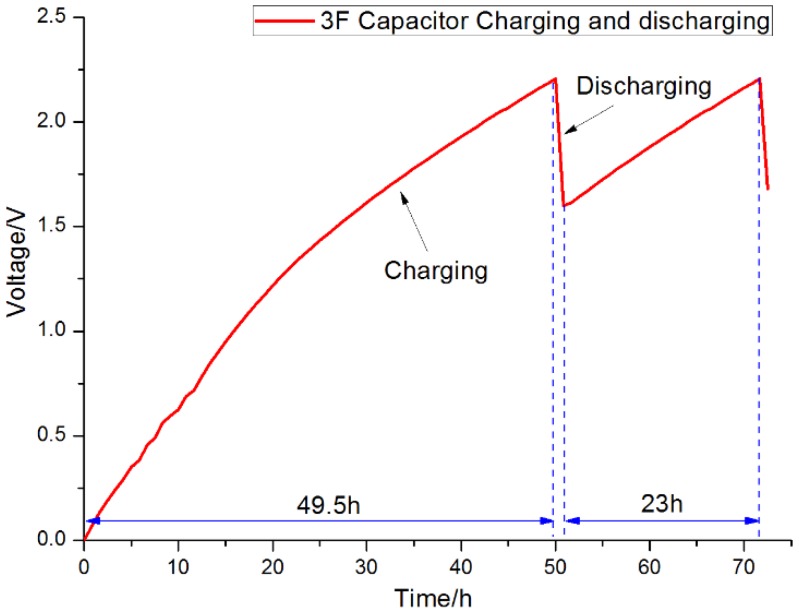
Charging and discharging curves of the 3 F supercapacitor.

Figure 12 shows that it required approximately 49.5 hours to increase the voltage from 0 to 2.2 V in the first period. The second period started at 1.6 V and charged to 2.2 V in 23 h, close to the time required during the first period to charge the voltage from 1.6 to 2.2 V. The discharging time required to reduce the voltage from 2.2 to 1.6 V is indicated in Figure 12 by the red dotted circle; this is when the voltage was boosted to 5 V to drive the load. The average current of MFC during charge period is about 0.2 mA. Supposing that Q1 is electrical charge produced from an individual MFC, Q2 is electrical charge stored in the supercapacitor, and E is the energy efficiency of the circuit, these values could be calculated as follows:

Q1 = I × t = 0.2 × 10^−3^ × 49.5 × 3600 s = 35.64 C
Q2 = C × U = 3 F × 2.2 V = 6.6 C
E = Q2 / Q1 = 6.6 / 35.64 = 18.518%

**Figure 13:**
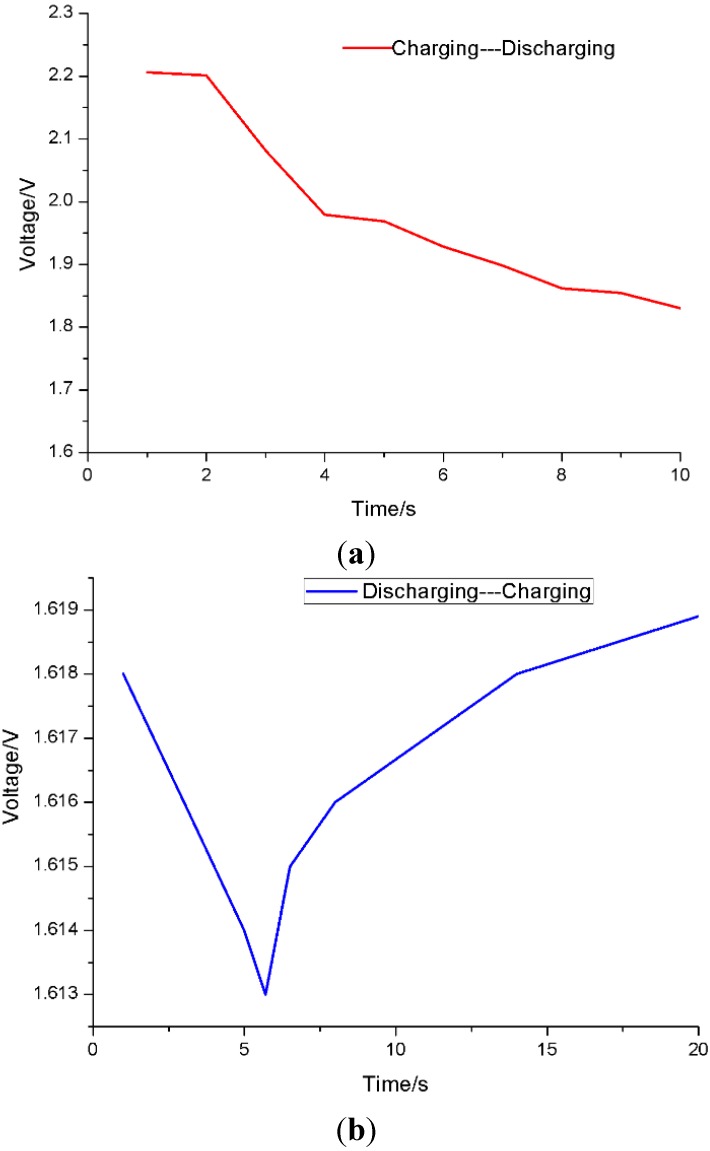
Turning points between charging and discharging and vice versa. (**a**) Turning points between charging and discharging; (**b**) Turning points between discharging and charging.

The efficiency of the boost converter is 85%–90% according to the datasheet of the boost converter [21]. So that the energy efficiency of designed PMS is about 15.7%–16.7%, higher than 5.33% reported on reference [25]. To illustrate this cyclical charging and discharging process in detail, the turning points between the charging and discharging of the supercapacitor, and *vice versa*, are shown in Figure 13. When the supercapacitor discharged, the curve fell immediately. On the contrary, the voltage rose quickly when the supercapacitor was in the charging state.

### 3.3. Application of the Designed PMS to Drive the Sensor and Telemetry System

**Figure 14:**
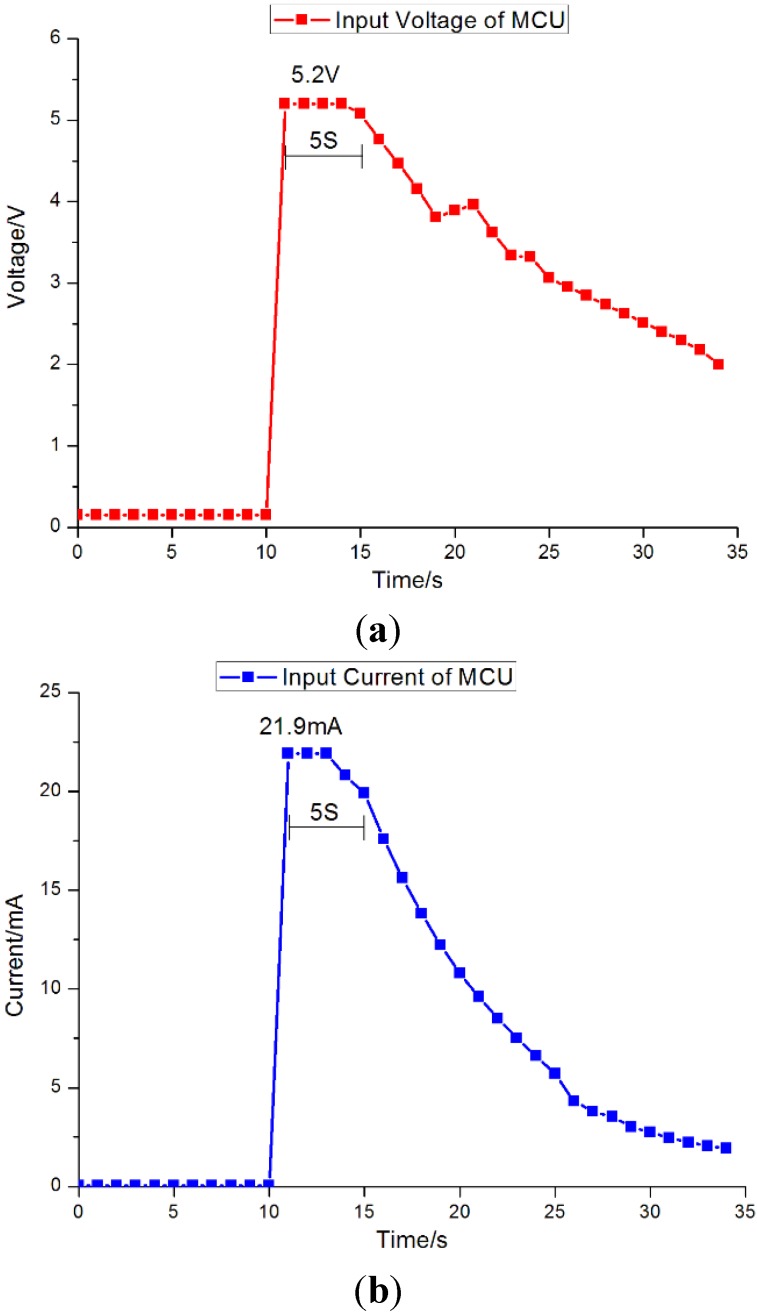
Voltage and current inputting the MCU. (**a**) Voltage inputting the MCU; (**b**) Current inputting the MCU.

The entire experimental setup consisted of an individual MFC, a collecting and storing energy circuit, and the wireless sensing transmitter. The 3 F supercapacitor stored the energy generated from the MFCs by means of the proposed PMS. The elevated voltage (5 V) discharged from the supercapacitor could be conducted as the power to the wireless sensor. The current and voltage inputting the MCU were 21.9 mA and 5.2 V, respectively; these input curves are shown in Figure 14. The operation time of the sensor depended on the storage capacity of the supercapacitor. In this case, the 3 F supercapacitor could power the sensor for 5 s.

**Figure 15:**
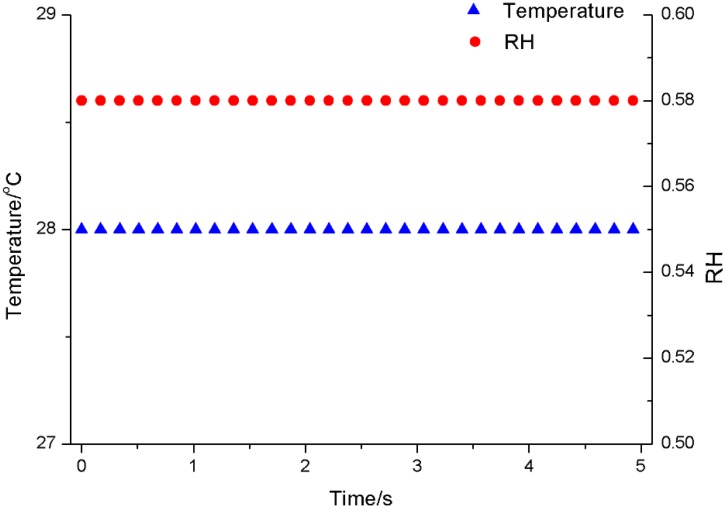
Received temperature and relative humidity data.

At the end of the receiver, the data emitted from the sensor could be accepted through serial ports. During the 5 s of the sensor’s operation, 30 data points were detected from the transmitter, and the results are shown in Figure 15. The temperature was 28 °C, and the relative humidity was 0.58, relative to vapor saturation in the air. At the same time, the system was shown to be capable of storing and displaying the data points using the serial port of the virtual instrument. 

From the test, it can be concluded that the application of the method–first for storing energy, and then for boosting voltage—enabled the actuation of the low-power devices with the intermittent output energy from the MFCs.

## 4. Conclusions

An energy harvesting circuit based on an individual MFC, a charge pump, and a supercapacitor has been implemented in this paper. The proposed PMS uses a supercapacitor to store energy to accumulate electricity. When the voltage at the supercapacitor’s node reaches 2.2 V, the charge pump produces a control signal allowing the switch to be on, and the supercapacitor starts to discharge. The discharge can be increased to 5 V with the help of a DC-DC boost circuit. The transmitter of the wireless sensing system can operate while capturing temperature and humidity data. The computer at the receiving end can store and display the data using serial ports. 

According to the testing performed in this study, the energy efficiency of designed PMS is about 15.7%–16.7%. The time interval between two samples is approximately 23 h. With the 3 F supercapacitor as the energy harvester, the sensor and transmitter system could work normally for 5 s, and 30 samples could be detected and transmitted during each sampling period. The time interval can be shortened if fewer samples are required from each sampling period. In theory, if only one sample was needed per period, the time interval could be reduced to approximately 50 min by using the 3 F supercapacitor as the energy harvester. 

MFCs are a promising technology that may contribute toward sustainable energy production and energy savings in wastewater treatment. However, the low power and voltage produced by an individual MFC cannot satisfy the demands of practical devices. Although multiple MFCs can be directly linked in series, the overall voltage of these cascaded MFCs cannot be sustainably increased because of voltage reversal, which decreases the stack voltage. This study certifies that it is feasible to use an individual MFC along with the designed PMS, as the power supply for a sensor and telemetry system that can collect one sample per hour. This sample period is useful for environmental monitoring where time based changes to temperature and humidly are small. In addition, with minor improvements to efficiency and cell capacity, this sample rate could easily approach 4–5 samples per hour.
